# Trajectories of allostatic load among older Americans and Britons: longitudinal cohort studies

**DOI:** 10.1186/s12877-018-0947-4

**Published:** 2018-10-23

**Authors:** Gindo Tampubolon, Asri Maharani

**Affiliations:** 1 Sociology and Cathie Marsh Institute for Social Research, University of Manchester, Humanities Bridgeford Street Building, Oxford Road, Manchester, M13 9PL UK; 2 Division of Neuroscience and Experimental Psychology, School of Biological Sciences, Faculty of Biology, Medicine and Health, University of Manchester, Manchester Academic Health Science Centre, Manchester, M13 9PL UK

**Keywords:** Allostatic load, Ageing, Longitudinal analysis, Attrition

## Abstract

**Background:**

Difference in life expectancy between males and females has been suggested to rest on sex difference in physiological dysregulation. But allostatic load, a physiological index, has not been carefully examined for an extended period beyond middle age. We aim to draw longitudinal trajectories of allostatic load in a national sample of older Americans and Britons; also to examine sex-based trajectories and factors behind their differences.

**Methods:**

We studied men and women aged ≥50 years participating in the Health and Retirement Study Waves 8–11, 2006–2012 (*N* = 15,583 person-years) and the English Longitudinal Study of Ageing Waves 2, 4 and 6, 2004–2012 (*N* = 14,765 person-years). Because of the difference in provenance, we included different number of biomarkers to calculate allostatic load in HRS and ELSA. In HRS we used 8 biomarkers (systolic and diastolic blood pressure, haemoglobin A1c, high-density lipoprotein, total cholesterol, waist circumference, cystatin C, and C-reactive protein), while ELSA allostatic load was constructed from 10 biomarkers (systolic and diastolic blood pressure, haemoglobin A1c, high-density lipoprotein, total cholesterol, waist circumference, BMI, triglyceride, fibrinogen and C-reactive protein). A growth curve model was fitted to repeated observations of allostatic load, demographic characteristics, socioeconomic position, comorbidities and health behaviours (smoking, drinking, and physical exercise). To account for attrition, a joint model was applied.

**Results:**

The analysis showed that allostatic load increases linearly with age in the U.S. However, there are different levels for males and females. In England allostatic load follows such different paths that their trajectories cross in later life.

**Conclusions:**

Sex-based trajectories of allostatic load showed distinct female advantage and are mostly consistent with female advantage in life expectancy.

## Background

The increasing proportion of older people in the population and their increasing life expectancy give larger number of older people longer lives [1]. This prospect raises the challenge of ensuring that the bulk of their lives is lived in a state of good health, free of morbidity and frailty, that will facilitate their continuing contributions to society, polity and the economy. Between the sexes, however, life expectancy differs with an advantage to females. Both in the US and the UK, government actuaries listed different life expectancies at various ages for males and females, with higher values for females. This advantage narrows down with age; thus sex difference in life expectancy converges with age [2, 3].

Despite the female advantage in life expectancy, they have higher morbidity rates and a declined quality of life than males in later life [4]. Literature has reported lower performance of older women on balance and gait tasks than men [5–7]. Older women also had higher prevalence of falling [8, 9] and the incidence of physical ability [10, 11] than their male counterparts. Sex differences in body composition, physical activity, and aerobic fitness are among the plausible explanations of these differences. Prior studies found that female respondents reported less habitual physical activity and had lower cardiorespiratory fitness than male respondents [12, 13], which may result in have greater adiposity and less lean mass among older women [14]. The paradoxical differences physical health among males and females may also related to underlying physiological dysregulation, particularly allostatic load [15]. Yang and Kozloski reported that allostatic load is higher for females throughout adulthood (17+ years), showing a female disadvantage in allostatic load. The female advantage in life expectancy sits uneasily with female disadvantage in allostatic load.

Allostatic load captures the notion that the human physiological system is not a fixed and stable system but a dynamically stable one, especially in the face of insults, hazards and injuries to various organ systems [16–21]. Allostatic load is the physiological wear and tear that the body sustains throughout the life course. Allostatic load has been found to longitudinally predict self-rated health, physical function, frailty and ultimately mortality in the US and UK [22, 23]. It also shows gradients along markers of socioeconomic position [23]. Allostatic load is therefore useful not only as a risk factor for morbidity, frailty and mortality, but also in understanding the mechanism of ageing throughout the life course [22].

Few studies have been devoted to uncovering the longitudinal pattern of allostatic load based on repeated measurements. As to socioeconomic gradient in allostatic load, what role the gradient plays in longitudinal change remains relatively uncharted. When examining longitudinal patterns of allostatic load in later life, a study must also contend with the problem of attrition: i.e. when participants are lost to follow up for various reasons, some to do with health and death. This attrition problem is pervasive in ageing studies [24], and to deal with this various models have been applied [25–29], including joint model as applied to the English Longitudinal Study of Ageing [25, 26].

To focus our efforts four questions will be answered. First, is there a sex difference in longitudinal trajectories of allostatic load? Second, given the female advantage in life expectancy, do these trajectories show a female advantage, i.e. a lower allostatic load trajectory for females? Third, given that psychosocial factors get under the skin and affect physiological regulation [20, 30, 31], what social factors are linked with the trajectories? Lastly, are there any differences between the two countries?

This work makes three contributions. It is the first to draw longitudinal trajectories of allostatic load for males and females based on the major study of ageing in America and Britain. Second, in drawing these trajectories, it has uncovered unique patterns that are consistent with the female advantage in life expectancy. Lastly, it showed perceptibly advantageous trajectories of allostatic load in older Americans consistent with emerging comparative research [32–34].

## Methods

We used two nationally longitudinal studies of aging: the U.S. Health and Retirement Study (HRS) [35] and The English Longitudinal Study of Ageing (ELSA) [36]. HRS is a multinational biannual household panel survey that collects information on the demographic characteristics, socioeconomic position, comorbidities and health behaviours of individuals aged 50+ in the U.S., while ELSA provided similar information among individuals in the same age range in England. HRS study was started in 1992, while the first wave of ELSA data was collected in 2002. So far there are twelve and seven waves of HRS and ELSA, respectively. This study used waves 8 to 11 of HRS (2006–2012) and every even-numbered wave (2004, 2008 and 2012/2013) of ELSA as the biomarker information was collected in those waves.

### Dependent variable

Due to the availability of the data, we included different numbers of biomarkers in HRS and ELSA. Eight biomarkers (systolic and diastolic blood pressure, haemoglobin A1c, high-density lipoprotein/total cholesterol, waist circumference, cystatin C, and C-reactive protein) in the HRS and ten biomarkers (systolic and diastolic blood pressure, haemoglobin A1c, high-density lipoprotein/total cholesterol, waist circumference, BMI, triglyceride, fibrinogen and C-reactive protein) in the ELSA were included in the analysis. Following Stephan et al. (2016) [37], we calculated allostatic load scores in HRS and ELSA in two stages. Firstly, we standardised each of the marker scores to have a mean of zero and a standard deviation of one. Secondly, we took the average of those standardised scores, resulting in a summary allostatic load scores that can be interpreted in terms of standard deviation units. Higher values indicate higher multisystem physiological dysregulation.

### Covariates

Demographic covariates include sex and age. Since age is capped at 90 in ELSA, information from respondents aged 50 to 89 in both surveys was used. Like other health functions, allostatic load is also shaped by social determinants of health. These include wealth tertiles (top, middle and bottom as reference), education (some college and high school or less as reference), and marital status (married/cohabiting and other as reference) [25, 38, 39].

Based on positive medical history (self-report of ‘has been diagnosed by professionals’), a sum of indicators about comorbidities were included, covering diabetes, cancer, hypertension, lung disease, heart condition, and stroke. Behavioural risk factors known to be important in other studies include smoking (current smoker versus not current smoker as reference), drinking (days in a week having a drink) and physical activity (rigorous, moderate and mild or less as reference) [40].

We included only observation of respondents with complete information on the allostatic load components and the covariates in HRS (*N* = 15,583 observations) and ELSA (*N* = 14,765 observations) in the analysis. Differences between those included and left out were tested using *t*-test (continuous variables) or χ^2^ test (categorical variables). At baseline, compared to those left out the analytic sample is younger (67 versus 73 year, *p* < 0.001 in the U.S and 65 versus 67 year, *p* < 0.001 in England), has fewer women (50% versus 57%; *p* < 0.001 in the U.S. and 53% versus 54%; *p* = 0.338 in England), and has lower levels of allostatic load (− 0.01 versus 0.008, *p* = 0.039 in the U.S. and 1.8 versus 2, *p* < 0.001 in England).

### Statistical analysis

To model how allostatic load changes over time using repeated observations, a growth curve model with random intercepts is applied. This model gives consistent estimates of the effects of risk factors when some participants are lost in subsequent visits, assuming attrition at random. We fit two models to address the questions above. First the main *allostatic* model built one trajectory for both sexes. Second, to check whether there is a difference in the rate of change between the sexes, the *interaction* model has an additional sex-age interaction term. To deal with attrition bias, a joint model is used [24, 26, 27, 41]. This model has recovered robust estimates of cognitive ageing in Australia [27], quality of life and cognition among older adults in England [25, 26]. We used the joint model to deal with the attrition in the longitudinal study. The random effects (*h(.)* below) in the joint model influence both allostatic load, *y*, and attrition, *t*, given these, allostatic load and attrition are independent. A joint model has two parts: the growth curve model (*f(.)* below) and the survival model (*g(.)* below)with gender, age polynomial of degree three and the random intercepts from the first partt [25, 26].

$$ L=\int f\left({y}_i|{b}_i,{x}_i\right)g\left({t}_i|{b}_i,{x}_i\right)h\left({b}_i\right)d{b}_i $$

We compare the results of growth curve models with those of joint or attrition-corrected models. The analyses were done in Stata 14 (StataCorp LP, College Station, Texas) and Latent Gold Syntax 5.1.

We carried out three supplementary analyses to check the robustness of our results. In the first supplementary analysis, we followed Read and Grundy (2014) by computing allostatic load score in ELSA using 13 indicators of whether biomarker values fell into their high risk quartiles [23]. The indicators cover five organ systems, including cardiovascular (diastolic blood pressure, systolic blood pressure), inflammation (CRP and fibrinogen), metabolic (haemoglobin A1c, high-density lipoprotein/total cholesterol ratio, triglycerides, and fasting glucose), body fat (BMI, waist circumference), and respiratory systems (forced expiratory volume, forced vital capacity and passive expired volume). Within each organ system each indicator receives the same proportion, for example the inflammation system has two biomarkers, each potentially contributing half a point when the biomarker value is found in the high risk quartile (zero otherwise) [22, 23]. The threshold of the high risk quartile is replaced with a clinical cut-off when there is one, for example the cut-off for male waist circumference is 102 cm. Finally, medication was considered, so that the value is automatically considered as high risk if the participant is using medication. The medication includes blood pressure lowering medication, anticoagulants, lipid lowering medication, diabetes medication and lung function medication. The scores range from 0 to 5.

In the second supplementary analyses, we analysed only the post-war cohort (those who were born on 1946 onwards) of ELSA to examine the effect of cohort on the allostatic load. Our previous work found that the trajectories of depressive symptoms between cohorts (pre-war, war, and post-war cohorts) in the US and England took different shapes [32]. The trajectories of pre-war cohorts in both countries and war cohort in England took a U-shape, while those of war cohort in England and post-war cohorts in both countries followed an inverted U-shape. We thus hypothesised that the allostatic load trajectory of the post-war cohort in ELSA follows that in HRS. In the final sensitivity analysis, we included the relevant medications taken by the respondents (medications for hypertension, hyperlipidaemia, and diabetes mellitus in ELSA and medication prescribed by the health professionals in HRS), and height (in cm).

## Results

The sample for analysis consists of 56% and 54% females in HRS and ELSA data, respectively (Table 1). The table also contains distribution across age groups, only for description and not for modelling, which shows that for either males or females there is an increasing age gradient in allostatic load in both data. In particular it shows that before 70 years in ELSA and before 80 years in HRS, females have lower allostatic load than males, but beyond this age the order changes. So although both sexes appear to have comparable levels of allostatic load, its age distribution is quite different in the two sexes.

**Table 1:** Descriptive summaries of allostatic load scores (means) in different groups

	HRS				ELSA			
	N	All *N* = 15,583	Male *N* = 6787	Female *N* = 8796	N	All *N* = 14,765	Male *N* = 6721	Female *N* = 8044
All		0.006	0.027	− 0.009		1.926	1.92	1.932
*Age group*								
50-	3752	− 0.044	0.006	− 0.084	4109	1.554	1.592	1.523
60-	4532	−0.002	0.025	−0.023	5843	1.834	1.850	1.821
70-	4735	0.028	0.035	0.023	3648	2.255	2.210	2.291
80-	2564	0.056	0.047	0.064	1165	2.676	2.553	2.771
*Married/cohabit*								
Not married	4753	0.047	0.067	0.04	4555	2.116	2.07	2.139
Married	10,830	−0.011	0.017	−0.041	10,210	1.842	1.876	1.807
*Wealth tertiles*								
Poorest	4891	0.069	0.098	0.049	4135	2.251	2.198	2.289
Middle	5303	0.001	0.024	−0.016	5089	1.97	1.968	1.971
Wealthiest	5389	−0.045	−0.025	− 0.062	5541	1.645	1.696	1.598
*Education*								
< High school	2569	0.077	0.087	0.069	4561	2.252	2.249	2.254
High school	5080	0.029	0.059	0.011	5692	1.884	1.922	1.855
Some college	7934	−0.031	− 0.007	− 0.051	4512	1.651	1.692	1.6
*Num. comorbidities*								
0	4160	− 0.121	− 0.086	−0.143	6401	1.304	1.306	1.302
1	5270	−0.000	0.011	−0.009	5667	2.247	2.238	2.254
2	3671	0.065	0.074	0.057	2016	3.044	2.634	2.585
3+	2482	0.148	0.136	0.161	681	3.193	3.202	3.187
*Current smoker*								
No	13,611	0.006	0.024	−0.007	12,979	1.902	1.889	1.912
Yes	1972	0.009	0.049	−0.022	1786	2.107	2.147	2.074
*Drinking*								
Less	14,001	0.008	0.031	−0.008	9098	2.057	2.054	2.059
Daily	1582	−0.007	0.001	−0.021	5667	1.716	1.763	1.66
*Vigorous physical activity*								
No	9994	0.042	0.07	0.024	12,026	2.027	2.037	2.02
Yes	5589	−0.057	− 0.03	− 0.086	2739	1.483	1.51	1.451

Married, better-educated and wealthy participants have lower allostatic load compared to reference categories in both data. Lastly (bottom block), to pick one behavioural factor, more physical activity inversely associates with allostatic load.

Table 2 collects the results of the growth curve and joint models using HRS and ELSA. The first model, labelled *Main*, showed that allostatic load increases with age in HRS (β = 0.003, *p* < 0.001), and ELSA (0.002, *p* < 0.001). To check for different trajectories for females and males, a sex-age interaction term was added in the *Interaction* model. This model gave the interaction terms positive and significant coefficients in both data, suggesting the trajectories are significantly different for females and for males.

**Table 2:** Models of age trajectories of allostatic load, coefficients and standard errors: main and interaction models. Source HRS wave 8–11 (2006–2012) and ELSA Wave 2, 4 and 6 (2004–2012)

	*HRS*		*ELSA*	
	*Main*	*Interaction*	*Main*	*Interaction*
Age	0.003 ± 0.000***	0.001 ± 0.000***	0.002 ± 0.000***	−0.0001 ± 0.000
Sex, Female	−0.104 ± 0.007***	− 0.251 ± 0.047***	− 0.093 ± 0.009***	− 0.376 ± 0.062***
Age*gender		0.002 ± 0.000***		0.004 ± 0.000***
Married/cohabit	−0.023 ± 0.008***	−0.019 ± 0.008**	−0.007 ± 0.009	−0.002 ± 0.009
Middle wealth	− 0.042 ± 0.008***	− 0.042 ± 0.008***	− 0.043 ± 0.008***	− 0.043 ± 0.008***
Wealthiest	− 0.068 ± 0.009***	− 0.067 ± 0.009***	−0.091 ± 0.009***	− 0.090 ± 0.009***
High school	− 0.006 ± 0.011	− 0.007 ± 0.011	− 0.056 ± 0.011***	− 0.053 ± 0.011***
Some college	− 0.047 ± 0.01***	−0.048 ± 0.01***	− 0.102 ± 0.012***	− 0.101 ± 0.012***
Num. comorbidities	0.093 ± 0.003***	0.093 ± 0.003***	0.033 ± 0.003***	− 0.043 ± 0.008***
Current smoker	− 0.032 ± 0.011***	− 0.031 ± 0.011***	− 0.011 ± 0.013	−0.011 ± 0.013
Past smoker	0.006 ± 0.007	0.008 ± 0.007	0.089 ± 0.014***	0.088 ± 0.014***
Drink > 5 days/week	− 0.008 ± 0.011	−0.008 ± 0.011	− 0.021 ± 0.007***	−0.021 ± 0.007***
Vigorous exercise	−0.076 ± 0.007***	− 0.076 ± 0.007***	− 0.044 ± 0.008***	− 0.044 ± 0.008***
Constant	−0.159 ± 0.03**	−0.079 ± 0.039**	− 0.001 ± 0.038	0.147 ± 0.050***

**Note:** Sig.: * = 10% or less; ** = 5% or less; *** = 1% or less

**Fig. 1:**
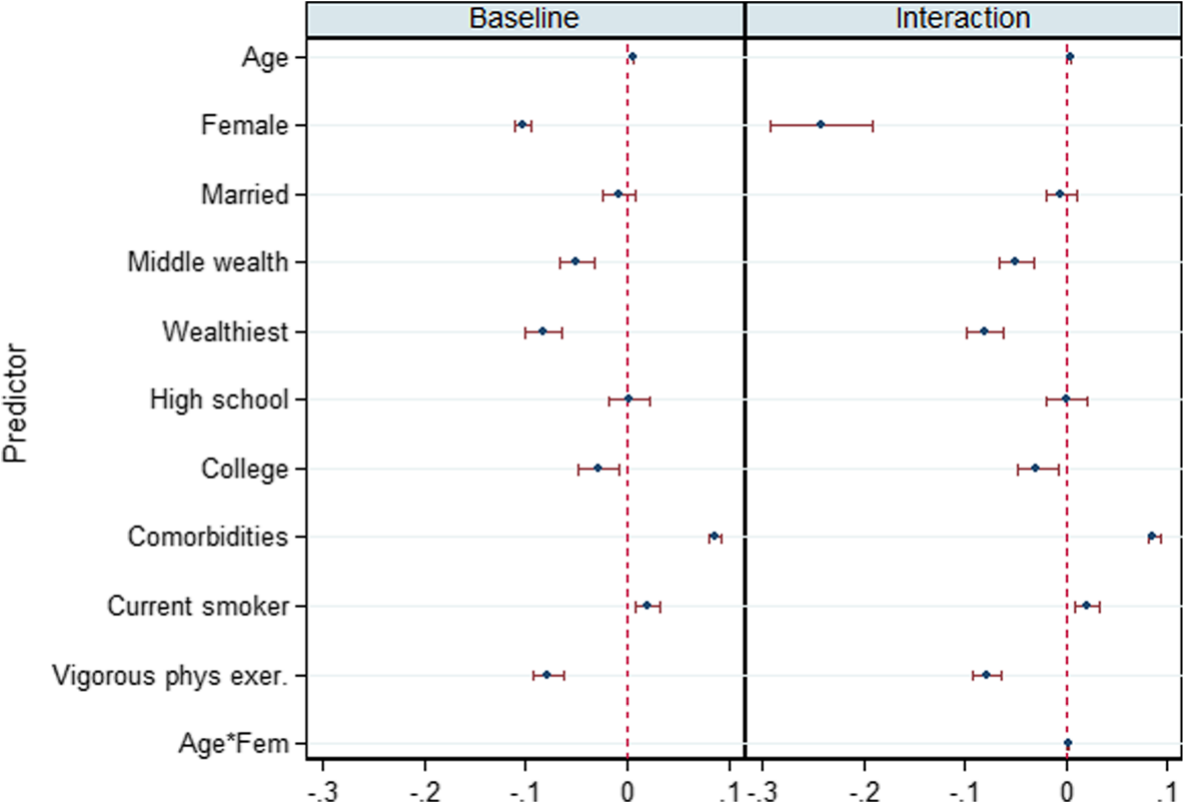
Attrition corrected models of age trajectories of allostatic load, coefficients and 95% confidence intervals: Main Allostatic and Sex interaction models. Source HRS 2004–2013

**Fig. 2:**
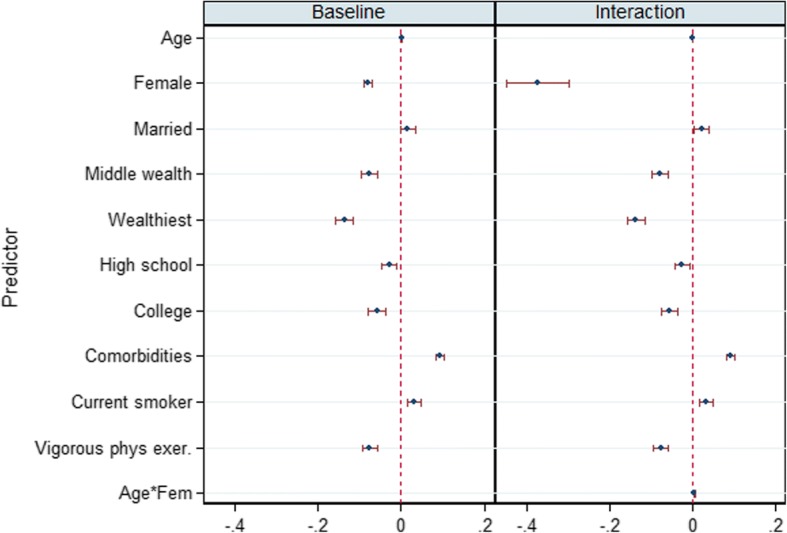
Attrition corrected models of age trajectories of allostatic load, coefficients and 95% confidence intervals: Main Allostatic and Sex interaction models. Source ELSA 2004–2013

To ease interpretation and comparison, the coefficients for the two joint models are plotted together in a figure of two panes corresponding to the two models (Fig. 1 for HRS and Fig. 2 for ELSA). Being female is associated with lower allostatic score in all models. The positive and significant coefficient for age and its interaction terms suggest different trajectories for females and for males. Other coefficients are not so different in the two survey data. Being wealthy and having high educational attainment have inverse relationships with allostatic score. The comorbidities and behavioural covariates (bottom block) also show similar patterns: comorbidities associate positively with allostatic load while rigorous physical exercise strongly associates with lower allostatic load.

**Fig. 3:**
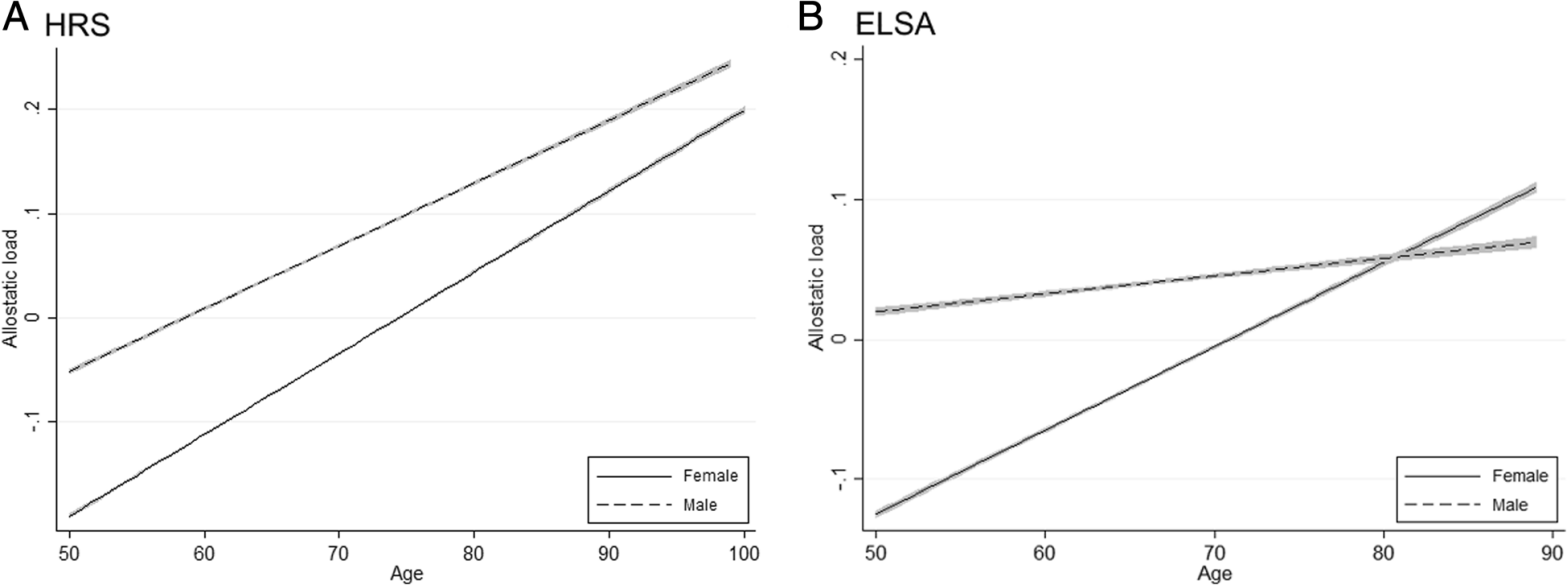
Predicted trajectories of allostatic load for females and males. Source HRS 2004–2013 and ELSA 2004–2013

Given that an extensive set of covariates is controlled, a summary of the trajectories based on the predicted allostatic load from the joint models can be drawn (Fig. 3). Comparing HRS (left) and ELSA (right) highlights striking similarities and differences. Older people on the west and east sides of the Atlantic accumulates allostatic load in a linear fashion as they age. In the US, females have lower allostatic load throughout later life, consistent with female advantage in life expectancy. A similar pattern characterises Britain although there is a crossover in the latter half when female advantage disappeared. Nonetheless, the sex difference throughout later life is slight and unlikely to be a clinically significant dysregulation [42]. Particularly the sex difference in Britain is negligible compared to that in America. Similar trajectories are found in our supplementary analyses using ELSA data. Being female is associated with lower allostatic load (β = − 0.451, *p* < 0.001) (see Appendix 1), we find a crossover of the allostatic load trajectories of males and females in the age of 57 (β = 0.005, *p* < 0.001) in which the allostatic load of the males is higher than that of females afterwards (see Appendix 2). The effect of cohort was shown in our analysis including only the post-war cohort of ELSA (see the Appendix 3). Supporting our previous work [32], the allostatic load trajectories of the post-war cohort of ELSA followed those of HRS: females have lower allostatic load throughout later life. The final sensitivity analysis demonstrated that the relationships between gender and allostatic load remained significant after controlling by height and the relevant medication (see Appendix 4). This sensitivity analysis shows that the results are robust.

## Discussion

We set out to draw the sex-based trajectories of allostatic load in samples representing the American and British older populations. Upward trajectories of allostatic load for both males and females are found, but the rates of change differ for the two sexes. This led to allostatic load incline among older Americans and additionally a cross-over in older British allostatic load trajectories at the mid-60s. Allostatic load trajectory as a physiological signature of ageing unfolds differently for males and females. This is a novel finding that deserves replication and reflection, some of which are offered later.

The patterns in America, i.e. linear accumulation and female advantage, are consistent with the patterns of life expectancy worldwide. These patterns also showed a gentler incline of allostatic load overtime, compared to those in Britain. The somewhat advantageous patterns revealed in America is consistent with recent evidence uncovered using the HRS and ELSA. Tampubolon and Maharani [32] showed that today older Americans are composed of sociohistorical cohorts (pre-war, war and post-war) that have an advantageous mental health. The majority (war and post-war cohorts) have advantageous trajectory depression in later life. Not so in older Britons: only the post-war cohort attains this advantage (ibid, Fig. 1). A larger proportion of older Americans than older Britons has a better mental health trajectory. Consistent with this, the HRS patterns on the left pane of Fig. 3 and the post-war cohort patterns on the Appendix 3 suggest a lower allostatic load trajectory among female than male at any age.

This study has a number of limitations. Direct measures of the stress response system such as urinary norepinephrine, epinephrine and salivary cortisol are not available. Including these is wholly desirable [18] but would no doubt be challenging. These older adult participants have given many physical and blood measurements over the years; therefore this study and others on allostatic load [23, 37] have tried to make the most of what the participants have already contributed. Another limitation is the handling of attrition. The joint model is only one solution; two other alternatives are pattern mixture model and selection models [24, 28]. Accounting for attrition with these alternatives is worth considering. Finally, one limitation of large samples is that the beta weights are very low, but the *p* values are statistically significant. Although there is a statistically significant change, the change is not strong.

An advantage of this study is the nationally representative nature of both HRS and ELSA, ensuring that the accumulation of physiological wear and tear in these populations can be uncovered without being unduly influenced by specific patterns in some clinical or patient samples. Another strength arises from its longitudinal design, with repeated measures not only of allostatic load but also of an extensive set of covariates.

The similar increase of allostatic load found among older Americans and older Britons stands in contrast with the trajectories found in American adults [15]. There the pattern is of female disadvantage and of divergence: females carry heavier allostatic load throughout adulthood and the difference continues to widen with age. The pattern was derived from cross-sectional data, and this may explain the contrast, since cross-sectional design has been found to give overestimates of changes in health functioning [43].

The socio-economic gradient in trajectories of allostatic load found here is consistent with results from an earlier study using a shorter period of the ELSA sample [23]. Similarly, the associations with health behaviours such as levels of physical exercise and smoking are consistent in the two studies. These behaviours regulate trajectories in comparable ways in both sexes. Our findings extend their results by including comorbidities, enabling net associations with socio-economic factors to be recovered.

## Conclusions

The trajectories of allostatic load in later life show distinct signatures for males and females. To uncover them not only is it necessary to use longitudinal data but also to account for attrition. With these done, both trajectories show clear social gradients in physiological wear and tear for an extended period in later life. Efforts to reduce these gradients earlier may yield fruit in later life by lightening the allostatic load, thus giving a healthier ageing experience to an increasingly large proportion of the population.

## Figures and Tables

**Fig. 4:**
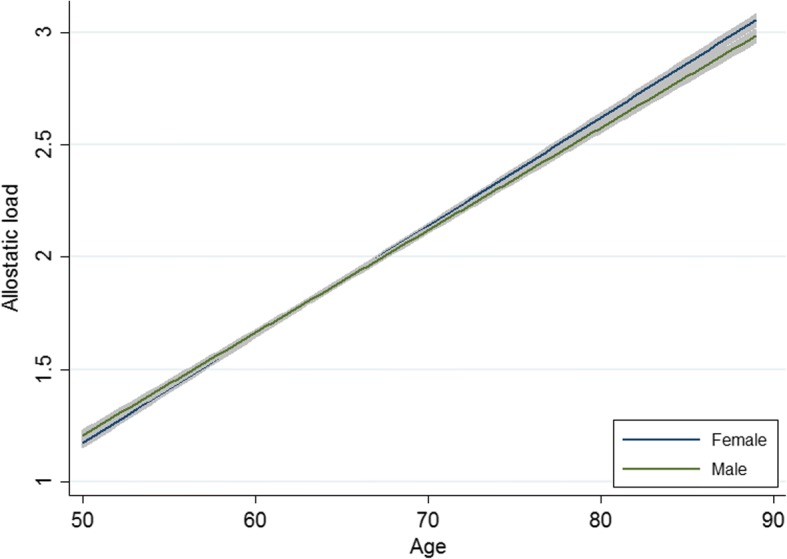
Predicted trajectories of allostatic load for females and males. Source ELSA 2004–2013. The Allostatic load score was calculated based on Read and Grundy (2014)

**Fig. 5:**
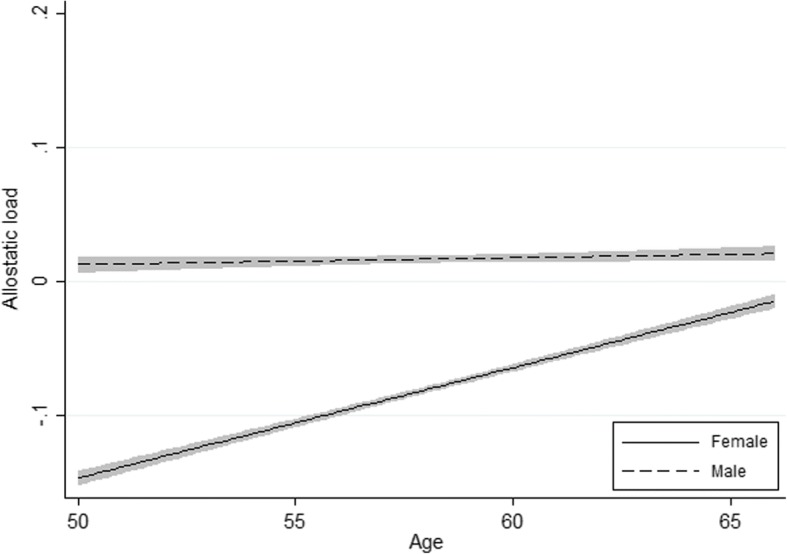
Predicted trajectories of allostatic load among post-war cohort for females and males. Source ELSA 2004–2013

## Appendix 1

**Table 3:** Models of age trajectories of allostatic load, coefficients and standard errors: main and interaction models. Source ELSA Wave 2, 4 and 6 (2004–2012). The Allostatic load score was calculated based on Read and Grundy (2014) [23]

	Main	Interaction
Age	0.036 ± 0.001***	0.032 ± 0.001***
Sex, Female	− 0.066 ± 0.018***	− 0.451 ± 0.126***
Age*gender		0.005 ± 0.001***
Married/cohabit	−0.027 ± 0.019	− 0.02 ± 0.019
Middle wealth	−0.16 ± 0.018***	− 0.159 ± 0.018***
Wealthiest	−0.304 ± 0.02***	− 0.303 ± 0.02***
High school	−0.086 ± 0.022***	− 0.083 ± 0.022***
Some college	−0.164 ± 0.025***	− 0.163 ± 0.025***
Num. comorbidities	0.389 ± 0.008***	0.39 ± 0.008***
Current smoker	0.125 ± 0.025***	0.126 ± 0.025***
Past smoker	0.004 ± 0.032	0.003 ± 0.032
Drink > 5 days/week	−0.102 ± 0.016***	−0.102 ± 0.016***
Vigorous exercise	−0.2 ± 0.017***	−0.201 ± 0.017***
Constant	−0.388 ± 0.077***	−0.185 ± 0.102

**Note:** Sig.: * = 10% or less; **: 5% or less; ***: 1% or less

## Appendix 4

**Table 4:** Models of age trajectories of allostatic load, coefficients and standard errors: main and interaction models. Source HRS wave 8–11 (2006–2012) and ELSA Wave 2, 4 and 6 (2004–2012)

	*HRS*		*ELSA*	
	*Main*	*Interaction*	*Main*	*Interaction*
Age	0.003 ± 0.000***	0.001 ± 0.000***	−0.000 ± 0.000	−0.002 ± 0.000***
Sex, Female	−0.071 ± 0.011***	−0.226 ± 0.048***	−0.110 ± 0.012***	− 0.408 ± 0.061***
Age*gender		0.002 ± 0.000***		0.004 ± 0.000***
Married/cohabit	−0.024 ± 0.008***	−0.020 ± 0.008**	−0.008 ± 0.009	−0.003 ± 0.009
Middle wealth	− 0.045 ± 0.008***	−0.044 ± 0.008***	−0.040 ± 0.008***	− 0.040 ± 0.008***
Wealthiest	−0.070 ± 0.009***	− 0.069 ± 0.009***	−0.083 ± 0.009***	− 0.082 ± 0.009***
High school	−0.006 ± 0.011	−0.008 ± 0.011	−0.054 ± 0.011***	−0.051 ± 0.011***
Some college	− 0.050 ± 0.010***	−0.050 ± 0.010***	−0.097 ± 0.012***	− 0.096 ± 0.012***
Num. comorbidities	0.093 ± 0.003***	0.094 ± 0.003***	0.033 ± 0.003***	− 0.033 ± 0.003***
Current smoker	−0.031 ± 0.011***	− 0.030 ± 0.011**	−0.006 ± 0.012	−0.006 ± 0.012
Past smoker	0.005 ± 0.007	0.007 ± 0.007	0.093 ± 0.014***	0.092 ± 0.014***
Drink > 5 days/week	−0.008 ± 0.011	−0.008 ± 0.011	− 0.015 ± 0.007**	−0.015 ± 0.007**
Vigorous exercise	−0.076 ± 0.007***	−0.076 ± 0.007***	−0.038 ± 0.008***	− 0.039 ± 0.008***
Height	0.216 ± 0.052***	0.218 ± 0.005***	−0.002 ± 0.000***	− 0.002 ± 0.000***
Taking prescribed medication	−0.007 ± 0.022	−0.008 ± 0.022		
Taking medication for hypertension			0.051 ± 0.008***	0.050 ± 0.008***
Taking medication for hyperlipidaemia			0.050 ± 0.009***	0.050 ± 0.009***
Taking medication for diabetes mellitus			0.207 ± 0.016***	0.209 ± 0.016***
Constant	−0.557 ± 0.102***	−0.476 ± 0.104***	0.464 ± 0.129***	0.616 ± 0.132***

**Note:** Sig.: * = 10% or less; ** = 5% or less; *** = 1% or less

## Abbreviations

BMI: Body Mass Index
CRP: C-Reactive Protein
ELSA: English Longitudinal Study of Ageing
HRS: Health and Retirement Study
UK: United Kingdom
US: United States
